# Integrative Multi-Omics Analysis Prioritizes Candidate Therapeutic Targets for Primary Open-Angle Glaucoma

**DOI:** 10.3390/ijms27114684

**Published:** 2026-05-22

**Authors:** Hao Kan, Lei Wen, Yuan Liu, Ka Zhang, Aiqin Mao, Li Geng, Fan Yu, Lei Feng

**Affiliations:** 1 Wuxi School of Medicine, Jiangnan University, Wuxi 214126, China; wenlei_9999@163.com (L.W.); yuan666l@163.com (Y.L.); zh0818k@163.com (K.Z.); maoaiqin110@126.com (A.M.); gengli@jiangnan.edu.cn (L.G.); jiangnanyf@126.com (F.Y.); feng2008lei@163.com (L.F.); 2 School of Food Science and Technology, Jiangnan University, Wuxi 214126, China

**Keywords:** primary open-angle glaucoma, Proteome-Wide Association Study, summary-data-based Mendelian Randomization, single-cell RNA sequencing

## Abstract

Primary open-angle glaucoma (POAG) is a leading cause of irreversible blindness driven by elevated intraocular pressure from compromised aqueous outflow. While genome-wide association studies have identified numerous risk loci, specific candidate proteins and their cellular mechanisms remain elusive. We employed a multi-omics framework integrating UK Biobank plasma proteomics (N = 53,022) and large-scale POAG GWAS summary statistics. We performed a Proteome-Wide Association Study, Mendelian Randomization, and Bayesian colocalization to infer causality. Identified candidates were mapped to human and mouse ocular scRNA-seq atlases to characterize cell-type specificity, followed by druggability assessments. We prioritized five putative causal proteins, with SEL1L and TFPI demonstrating the strongest evidence. Cross-species scRNA-seq revealed that SEL1L and SERPINF1 are robustly expressed in the trabecular meshwork (TM), particularly the juxtacanalicular tissue, implicating them in outflow resistance. Conversely, TFPI and SLC9A3R2 localize to Schlemm’s canal endothelium, suggesting a role in modulating barrier function. Pathway analyses highlighted endoplasmic reticulum protein processing and coagulation cascades. This study maps putative causal POAG proteins to conventional outflow pathway cells, highlighting SEL1L as a novel target for TM homeostasis and TFPI for drug repurposing, thereby providing data-driven hypotheses to facilitate precision glaucoma therapeutics.

## 1. Introduction

Primary open-angle glaucoma (POAG) affects nearly 70 million people worldwide and remains a leading cause of irreversible blindness [1]. The disease is characterized by progressive optic neuropathy, often associated with elevated intraocular pressure (IOP) due to increased resistance in the conventional aqueous humor outflow pathway [2]. Despite the availability of IOP-lowering therapies, a significant proportion of patients continue to experience vision loss, highlighting the urgent need to decipher the molecular pathology governing outflow resistance and to identify novel therapeutic targets [3].

Genome-Wide Association Studies (GWAS) have revolutionized our understanding of POAG susceptibility, uncovering over 100 genomic loci associated with the disease [4,5,6]. However, translating these genetic signals into biological mechanisms presents a major bottleneck. Most risk variants reside in non-coding regions, making the identification of effector genes and downstream proteins challenging [7]. Proteins, being the ultimate effectors of biological processes and the targets of over 90% of approved drugs, offer a direct bridge between genetic variation and clinical phenotypes.

The emergence of large-scale proteomic quantitative trait loci (pQTL) resources, such as the UK Biobank Pharma Proteomics Project (UKB-PPP) [8], provides an unprecedented opportunity to interrogate the genetic regulation of the human proteome. By integrating pQTL data with disease GWAS summary statistics, Proteome-Wide Association Studies (PWAS) and Mendelian Randomization (MR) approaches can prioritize proteins with putative causal relevance to disease etiology, distinguishing them from mere bystanders.

In this study, we adopted a comprehensive multi-omics framework to prioritize putative candidate proteins for POAG, aiming to advance the current mechanistic understanding of the disease through three key refinements. First, to ensure robust statistical power, we integrated the latest high-throughput plasma proteomics from the UKB-PPP (Olink Explore 3072) with the recent FinnGen R12 POAG GWAS data via PWAS, SMR, and colocalization analyses. Second, to overcome the limitation of using systemic plasma data to infer local ocular biology, we characterized our findings using cross-species (human and mouse) high-resolution single-cell transcriptomic atlases. While previous multi-omics investigations in POAG have often focused broadly on retinal and optic nerve tissues, our study specifically concentrates on the conventional aqueous humor outflow pathway—the physiological ‘bottleneck’ of IOP regulation. This approach allowed us to computationally bridge systemic plasma signals to high-resolution cellular niches, such as the cribriform juxtacanalicular tissue (JCT) and Schlemm’s canal endothelium. Third, through this targeted framework, we prioritize SEL1L as a novel, highly localized protective candidate for TM homeostasis—a mechanism not extensively detailed in previous POAG genetic studies. By mapping candidate expression to these highly specialized cell types, we provide a precise mechanistic context regarding their roles in outflow resistance, ultimately delivering a data-driven target prioritization roadmap to guide future experimental validation and facilitate precision glaucoma therapeutics.

## 2. Results

### 2.1. PWAS Identifies Distinct POAG-Associated Plasma Proteins

**Figure 1:**
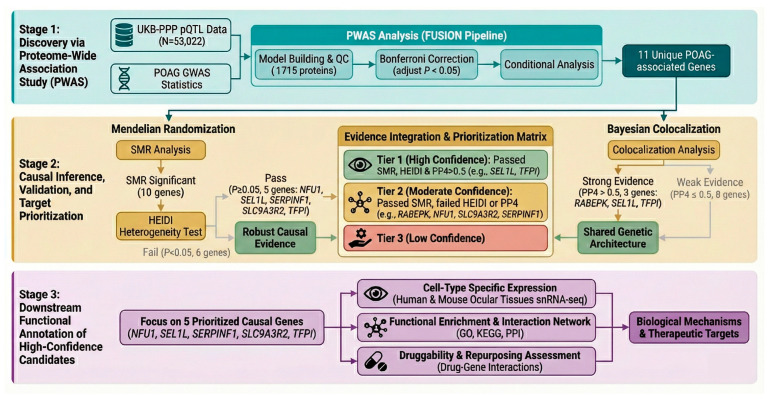
Multi-stage integrative framework for identifying and prioritizing putative causal candidate proteins for POAG.

**Figure 2:**
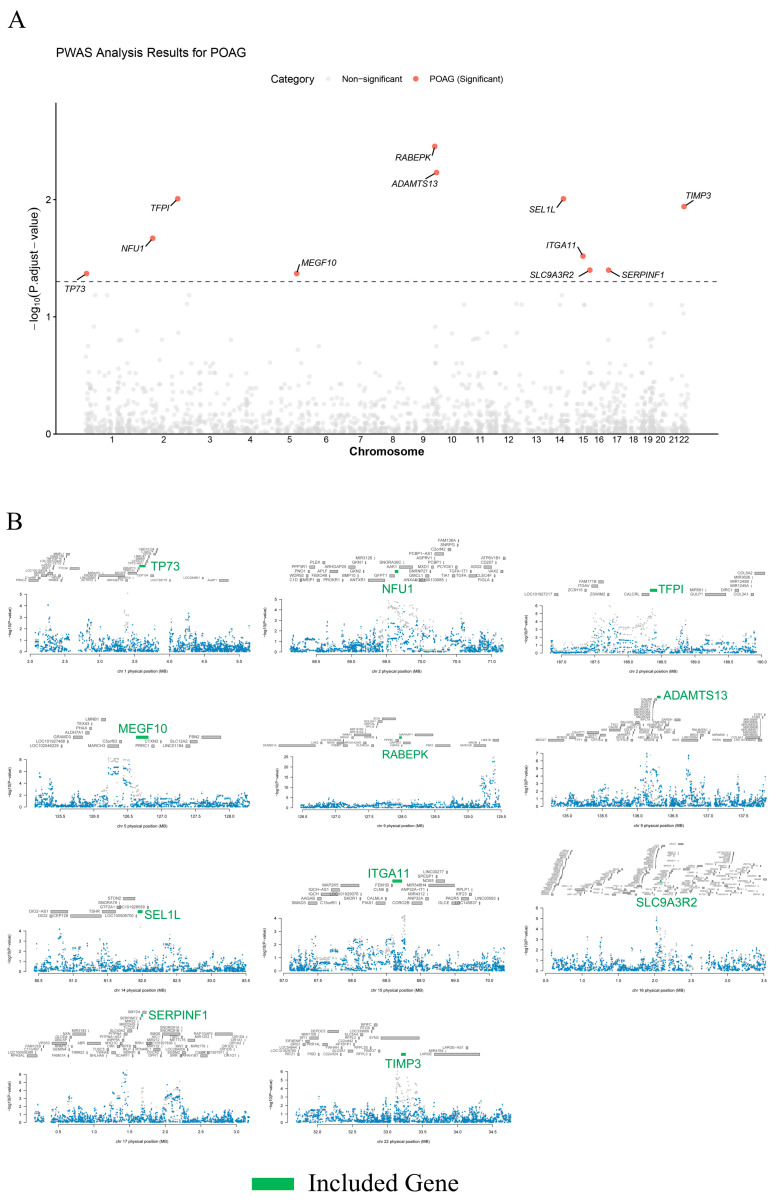
Results of the PWAS analysis. (**A**) Manhattan plot for the PWAS results for POAG. The x-axis represents the chromosomal position, and the y-axis indicates the significance level (−log_10_*P* adjusted value). Significant genes passing the Bonferroni correction threshold (dashed line) are highlighted in red and labeled with gene symbols. Gray dots represent non-significant associations. (**B**) Regional association plots for the identified risk loci following conditional analysis. The plots visualize the prioritization of causal genes within significant genomic regions, where blue dots represent individual single nucleotide polymorphisms (SNPs) plotted by their chromosomal positions (x-axis) and association significance (−log_10_*p*-value, y−axis). Genes highlighted in green (Included Gene) represent the prioritized candidates included in the credible set.

The overall study design is illustrated in Figure 1. We leveraged human plasma pQTL data from the UKB-PPP cohort (N = 53,022) to construct prediction models for 2923 proteins. After rigorous quality control, 1715 proteins exhibiting significant SNP-based heritability (*p* < 0.05, h^2^ > 0, Supplementary Table S1) were integrated with POAG GWAS summary statistics using FUSION (GitHub repository version accessed in April 2026) (Supplementary Table S2). This identified 11 significant protein-POAG associations (Bonferroni-corrected *p* < 0.05; Figure 2A, Table 1 and Supplementary Table S3). All 11 loci passed stepwise conditional analysis, confirming them as independent genetic signals associated with POAG (Figure 2B and Supplementary Table S4).

### 2.2. SMR and Colocalization Analyses Prioritize High-Confidence Causal Candidates

**Figure 3:**
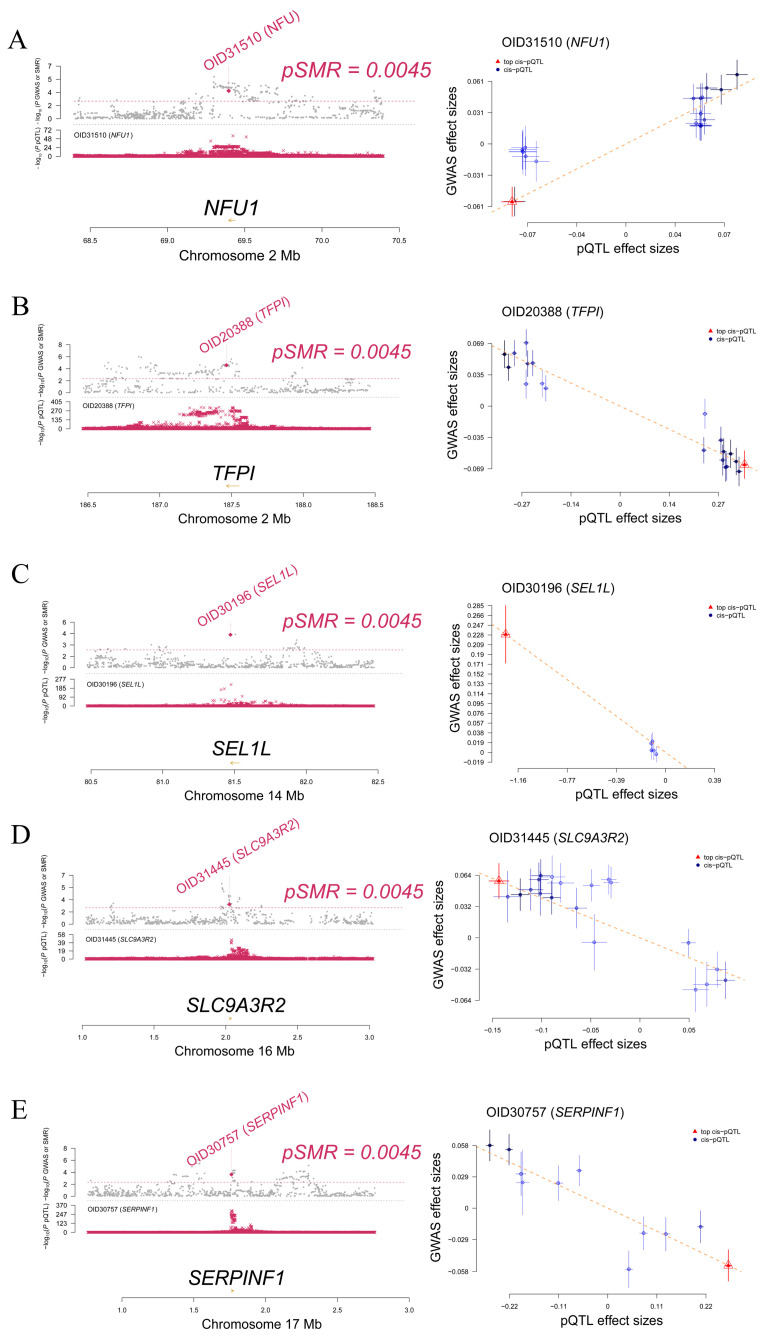
Results for the causal genes. SMR results for five putative causal proteins (**A**) NFU1, (**B**) TFPI, (**C**) SEL1L, (**D**) SLC9A3R2, and (**E**) SERPINF1. Left panels: regional association plots showing the colocalization of GWAS signals for POAG (**top plot**, gray dots) and cis-pQTL signals for the respective plasma protein (**bottom plot**, maroon dots). The x-axis represents the genomic position, and the y-axis represents the −log_10_(*p*-value). The top SMR SNP is highlighted with a diamond. Right panels: effect size plots visualizing the relationship between the genetic effect on protein abundance (x-axis, cis-pQTL beta) and the genetic effect on POAG risk (y-axis, GWAS beta). Each point represents a genetic instrument (SNP). The orange dashed line indicates the estimated causal effect (slope) from the SMR test. Error bars denote standard errors. Blue dots represent cis-pQTLs, and the red triangle highlights the top cis-pQTL instrument.

**Figure 4:**
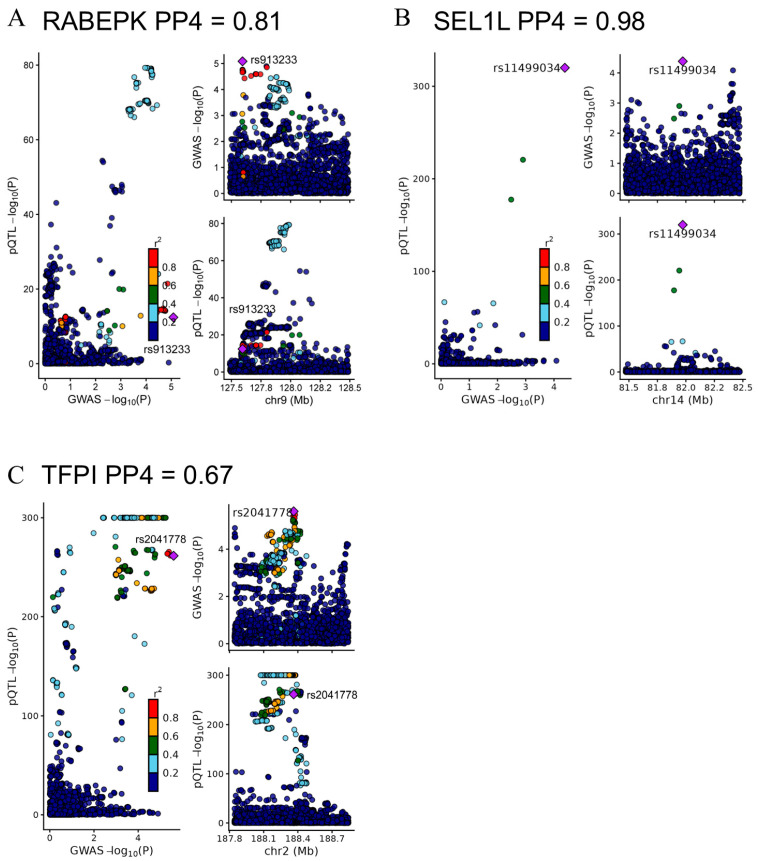
Bayesian colocalization analysis of POAG GWAS signals and plasma pQTLs. LocusCompare plots visualizing the colocalization events for three significant genes (**A**) RABEPK, (**B**) SEL1L, and (**C**) TFPI. For each panel, the left plot shows the correlation between the significance of the GWAS association (y-axis, −log10P) and the pQTL association (x-axis, −log10P) for all variants in the region. The right plots display the regional Manhattan plots for GWAS (**top**) and pQTL (**bottom**) data. The purple diamond represents the lead SNP for each locus (labeled with rsID). Other SNPs are colored based on their linkage disequilibrium (r2) with the lead SNP. The posterior probability for a shared causal variant (PP4) is indicated above each panel, with a threshold of PP4 > 0.5 considered strong evidence of colocalization.

To further assess putative causality, we applied SMR analysis to the 11 PWAS-identified candidates. Ten proteins (excluding ITGA11) showed significant correlations (Supplementary Table S5); however, the HEIDI test revealed that associations for 6 genes were likely driven by linkage disequilibrium (*P*_HEIDI_ < 0.05). Consequently, five proteins—NFU1, SEL1L, SERPINF1, SLC9A3R2, and TFPI—passed both thresholds, providing robust genetic evidence for a putative causal role (Figure 3A–E and Table 1). Crucially, evaluating the effect sizes (Beta values) provided directionality to these associations; for instance, SEL1L (β_SMR_ = −0.18) and TFPI (β_SMR_ = −0.19) exhibited negative effects, indicating that genetically predicted higher plasma levels of these proteins are associated with a reduced risk of POAG (i.e., acting as protective factors). Furthermore, rigorous Reverse MR analyses (leveraging 32 independent genome-wide significant SNPs as instruments) demonstrated that genetic liability to POAG does not secondarily drive alterations in the plasma levels of these five proteins (all Inverse Variance-Weighted *p* > 0.05; Supplementary Table S6). While these findings significantly reduce the concern for reverse causation, they cannot completely exclude it due to inherent limitations in instrument strength and statistical power. Bayesian colocalization analysis further refined these findings, providing strong probabilistic evidence that three proteins (RABEPK, SEL1L, and TFPI) shared a single causal variant with POAG (*PP4* > 0.5, Figure 4A–C). Notably, this strict classification highlighted methodological conflicts for certain loci. For example, RABEPK exhibited strong colocalization (*PP4* = 0.81) but failed the HEIDI test (*p* = 3.18 × 10^−3^). This conflict indicates a violation of colocalization’s single-causal-variant assumption, suggesting the presence of multiple causal variants or horizontal pleiotropy. Consequently, based on cumulative evidence, we classified the candidates into three tiers: Tier 1 (High confidence: SEL1L, TFPI), Tier 2 (Moderate confidence: RABEPK, NFU1, SLC9A3R2, SERPINF1), and Tier 3 (Low confidence: ADAMTS13, TIMP3, ITGA11, MEGF10, TP73) (Table 1).

### 2.3. scRNA-Seq Contextualizes Candidate Proteins Within the Conventional Outflow Pathway

**Figure 5:**
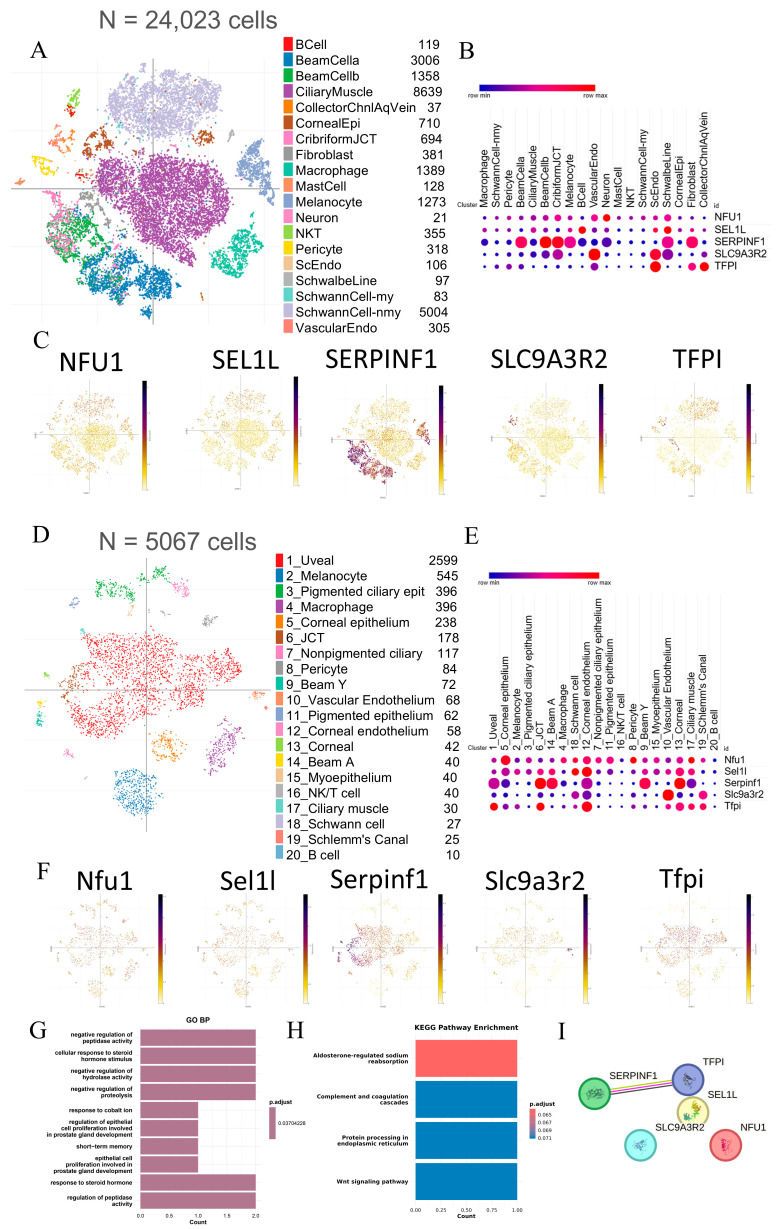
Single-cell transcriptomic profiling and functional characterization of putative POAG causal genes. (**A**–**C**) Expression analysis in the human ocular tissues. (**A**) UMAP of 24,023 cells from the human ocular tissues, colored by major cell class. (**B**) Dot plot summarizing the expression of the five prioritized genes (NFU1, SEL1L, SERPINF1, SLC9A3R2, TFPI) across human ocular cell types. The dot size represents the percentage of cells expressing the gene, and the color intensity (blue to red) indicates the average scaled expression level. (**C**) Feature plots visualizing the spatial expression patterns of the candidate genes on the human UMAP embedding. (**D**–**F**) Cross-species validation in the mouse ocular tissues. (**D**) UMAP visualization of 5067 cells from mouse ocular tissues, showing consistent clustering of JCT, Beam cells, and Schlemm’s canal. (**E**) Dot plot and (**F**) feature plots showing the expression of murine orthologs (Nfu1, Sel1l, Serpinf1, Slc9a3r2, Tfpi) in mouse ocular cell lineages. (**G**–**I**) Functional enrichment and interaction networks. (**G**) Gene Ontology enrichment analysis for Biological Processes. (**H**) KEGG pathway enrichment analysis. Bar lengths represent gene counts, and colors indicate statistical significance (*P*-adjust). (**I**) Protein–Protein Interaction network constructed from the STRING database. Nodes represent proteins, and edges represent known functional or physical interactions. The network highlights a strong interaction between SERPINF1 and TFPI, while other proteins operate as independent functional units.

To characterize the cell-type specificity and evolutionary conservation of the prioritized targets (NFU1, SEL1L, SERPINF1, SLC9A3R2, TFPI), we mapped their expression using high-resolution scRNA-seq atlases of human and mouse ocular tissues (Figure 5A–F). In the human dataset (N = 24,023 cells), SEL1L and SERPINF1 exhibited striking spatial enrichment in TM-associated clusters, specifically in beam cells and cribriform JCT (Figure 5B). This localization—within the primary site of outflow resistance—suggests a direct role in modulating TM stiffness. Conversely, TFPI and SLC9A3R2 were predominantly restricted to endothelial lineages (e.g., Schlemm’s canal endothelium), implying involvement in endothelial barrier maintenance. Crucially, murine orthologs recapitulated these human expression signatures with high fidelity (Figure 5E,F), reinforcing the functional conservation of these proteins in IOP regulation.

### 2.4. Functional Enrichment Highlights ER Processing and Coagulation Cascades

GO analysis indicated significant enrichment in the regulation of peptidase and hydrolase activity, processes critical for extracellular matrix (ECM) remodeling within the TM (Figure 5G and Supplementary Table S7). KEGG pathway analysis provided compelling mechanistic insight: “Protein processing in endoplasmic reticulum” was significantly enriched (Figure 5H), corroborating our scRNA-seq mapping of SEL1L to TM cells and underscoring ER stress modulation as a protective mechanism against IOP elevation. Additionally, enrichment in “Complement and coagulation cascades” aligns with TFPI’s endothelial localization. PPI network analysis revealed a direct functional interplay between SERPINF1 and TFPI (Figure 5I and Supplementary Table S8), suggesting a coordinated mechanism in vascular homeostasis, while SEL1L, SLC9A3R2, and NFU1 appeared to operate through independent biological pathways.

### 2.5. Druggability Assessment Identifies Repurposing and De Novo Targets

Interrogation of DGIdb and DrugBank revealed that TFPI possesses the highest druggability profile, targeted by approved biologics like Andexanet alfa and Concizumab (primarily for bleeding disorders), highlighting it as an immediate candidate for future repurposing evaluation. SLC9A3R2 interacts with investigational small molecules (e.g., CI-1040), offering chemical probes for mechanistic studies. Conversely, SEL1L remains ‘undruggable’ in current databases, presenting a novel, previously uncharacterized de novo target for therapeutic development focused on TM cell homeostasis (Supplementary Table S9).

## 3. Discussion

In this study, we bridged the gap between genetic discovery and biological mechanism in glaucoma by integrating large-scale plasma proteomics with localized single-cell transcriptomics. Ultimately, we identified five proteins genetically associated with POAG. Crucially, we mapped their expression to distinct cellular niches within ocular tissues.

Our most compelling finding is the identification of SEL1L as a high-confidence protective factor. SEL1L is a core component of the ER-associated degradation (ERAD) complex, which is critical for clearing misfolded proteins [9,10]. The trabecular meshwork faces significant mechanical stress and is highly prone to misfolded protein accumulation. This accumulation subsequently induces ER stress and increases outflow resistance [11,12]. Notably, our single-cell annotation specifically localized SEL1L to the cribriform JCT and beam cells.

**Figure 6:**
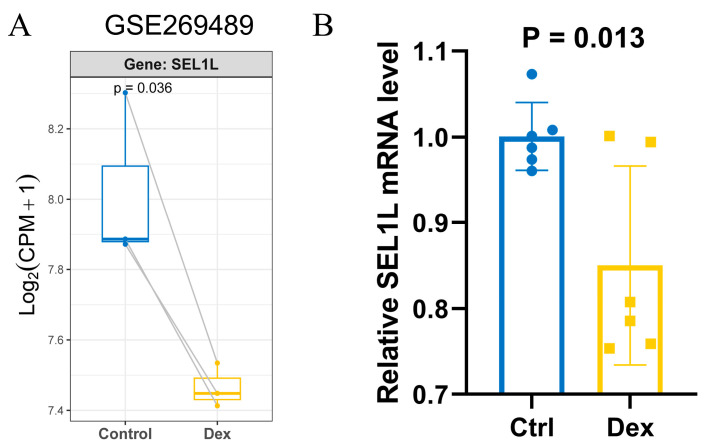
Validation of SEL1L expression in in vitro models. (**A**) Validation of SEL1L expression in an independent in vitro model. Paired analysis of primary human trabecular meshwork cells (HTMCs) treated with Dexamethasone (Dex) versus DMSO control (Dataset: GSE269489). SEL1L is significantly downregulated under Dex-induced pathological stress (Paired *t*-test, *p* = 0.036). (**B**) Relative mRNA expression levels of SEL1L in ARPE-19 cells in vitro. Cells were treated with control or 1 μM Dexamethasone (Dex) (N = 6 per group). SEL1L mRNA levels are significantly decreased following Dex treatment (*p* = 0.013).

To transcend purely computational hypotheses, we evaluated SEL1L expression in in vitro models. First, we analyzed an independent transcriptomic dataset (GSE269489). Paired analysis revealed a significant downregulation of SEL1L in human primary TM cells under dexamethasone-induced pathological stress (*p* = 0.036, Figure 6A). Furthermore, our independent in vitro experiments demonstrated that dexamethasone treatment significantly decreased SEL1L mRNA levels in ARPE-19 cells (*p* = 0.013, Figure 6B). We acknowledge that ARPE-19 cells, derived from the retinal pigment epithelium, are not direct surrogates for TM or Schlemm’s canal biology. Therefore, this validation primarily supports the general stress-response relevance of SEL1L under pathological conditions, rather than providing direct mechanistic confirmation within conventional outflow tissues. Nevertheless, this active pathological depletion strongly supports our genetic finding that basal SEL1L is protective. It suggests that cells with inherently higher SEL1L capacity are better buffered against ER stress-induced TM dysfunction. Although targeted therapeutics for SEL1L are currently lacking, it remains a mechanistically supported candidate for enhancing TM health. To bridge the translational gap for targets lacking experimental structural data, recent advances in AI-based protein structure prediction (e.g., AlphaFold) offer a highly practical framework for future structure-based drug design [13,14,15].

Beyond SEL1L, we identified TFPI (Tissue Factor Pathway Inhibitor) as a key protective factor enriched in the Schlemm’s canal (SC) endothelium. The SC functions similarly to a lymphatic vessel, with its inner wall endothelium forming the final barrier to aqueous outflow [16]. As a potent anticoagulant and anti-inflammatory protein [17], TFPI may play a critical dual role. These findings provide exploratory, hypothesis-generating insights suggesting that TFPI prevents local microthrombosis in the distal outflow tract while concurrently maintaining endothelial barrier integrity. Interestingly, TFPI is a ‘druggable’ target with approved biologics (e.g., Andexanet alfa and Concizumab). However, these existing drugs are inhibitors used clinically to promote coagulation. In contrast, our genetic data (Beta < 0) dictates that higher levels of TFPI protect against POAG. This presents a ‘directionality paradox’ for direct drug repurposing. Administering existing TFPI inhibitors would theoretically exacerbate glaucoma risk. Therefore, successfully translating TFPI into a glaucoma therapeutic requires an opposing pharmacological strategy. Future efforts should focus on developing novel TFPI agonists or localizing the delivery of recombinant TFPI to mimic its natural protective effect on the SC barrier.

Additionally, SERPINF1 (PEDF) was prominently enriched in the TM [18,19]. Known for its neuroprotective and anti-angiogenic properties, PEDF may inhibit fibrotic remodeling in the outflow pathway. However, further in vivo functional assays are required to definitively validate these exploratory mechanistic hypotheses. Finally, SLC9A3R2 (NHERF2), a scaffold protein regulating ion exchangers [20,21], was localized to the endothelium. This suggests a potential role in influencing the ionic composition and fluid dynamics of the aqueous humor.

### Limitations and Future Directions

Several limitations must be acknowledged. First, given our current sample size of 10,832 POAG cases, the statistical power to detect modest effects (e.g., OR ~1.1 per standard deviation of plasma protein) remains limited. Consequently, some null findings in our pipeline may represent Type II errors rather than true biological negatives. Second, our strict adherence to a cis-only MR design systematically underestimates the contribution of trans-regulated targets, despite being highly effective at minimizing horizontal pleiotropy. This trade-off is particularly notable for plasma proteins like SEL1L, whose circulating levels may be largely trans-determined by hepatic secretion. Third, complex loci such as RABEPK (an essential regulator of endosomal trafficking) displayed conflicting statistical signals. Specifically, RABEPK passed colocalization but failed the HEIDI test. While its biology is relevant to TM vesicle transport, this conflict suggests the presence of multiple causal variants. This reinforces the necessity of our strict tiering system. Fourth, inferring local ocular biology from systemic plasma pQTLs introduces inherent “tissue mismatch” uncertainty. It remains challenging to definitively distinguish between local ocular protein leakage into the systemic circulation versus systemic-to-local downstream effects. Reverse MR significantly reduced the concern for systemic changes secondary to POAG, and scRNA-seq provided a critical contextual filter. Nevertheless, future investigations utilizing aqueous humor proteomics are essential. Fifth, our MR findings support associations consistent with causal effects under strict instrumental variable assumptions. We provided independent in vitro transcriptomic validation demonstrating the pathological depletion of SEL1L. However, definitive causal claims ultimately require in vivo experimental perturbations in ocular tissue. Finally, the UKB-PPP and FinnGen datasets utilized in this study are predominantly derived from individuals of European ancestry. The genetic architecture of POAG exhibits well-documented differences across diverse populations. Therefore, our current findings necessitate future replication in diverse, multi-ancestry cohorts to ensure global applicability.

## 4. Materials and Methods

### 4.1. Data Sources

Plasma pQTL summary statistics were obtained from the UK Biobank Pharma Proteomics Project (UKB-PPP), which measured 2923 proteins in 53,022 individuals using the Olink Explore 3072 platform. Genetic summary statistics for POAG susceptibility were obtained from the FinnGen consortium’s 12th data release (R12) (https://www.finngen.fi/en, accessed on 19 May 2026). Within the FinnGen cohort, POAG cases were identified based on the International Classification of Diseases (ICD-10) code H40.1. Case status was further confirmed through linkage to national health registries, which included records of intraocular pressure-lowering medication prescriptions and glaucoma-related surgical procedures. Control subjects were defined as individuals with no registered diagnosis of glaucoma, ocular hypertension, or related optic neuropathies. The genome-wide association analysis conducted by FinnGen on this phenotypically curated dataset produced the summary statistics utilized in this study.

### 4.2. Proteome-Wide Association Study (PWAS)

PWAS was conducted using the FUSION software pipeline (GitHub repository version, updated as of April 2026) [22]. We constructed predictive models for plasma protein levels using robust SNPs from the UKB-PPP reference panel. Weights were computed using Best Linear Unbiased Prediction (BLUP, v1.68), LASSO v5, or Elastic Net v1.5.2 [23], with the FUSION pipeline automatically selecting the most robust predictive model for each protein based on the highest out-of-sample cross-validation *R*^2^ (requiring cross-validation *p* < 0.05). The association between genetically predicted protein levels and POAG risk was tested, with significance defined as a Bonferroni-corrected *p* < 0.05 (i.e., a nominal *p* < 2.91 × 10^−5^, correcting for 1715 proteins). To identify independent causal signals within shared genomic loci, we performed stepwise conditional analysis using GCTA-COJO. A linkage disequilibrium (LD) r^2^ threshold of <0.1 within a 1 Mb window was used to distinguish distinct genetic signals.

### 4.3. Causal Inference: SMR and Colocalization Analysis

To infer potential causality, we employed Summary-data-based Mendelian Randomization (SMR) (version 1.32) [24,25]. To minimize potential bias from horizontal pleiotropy and ensure the biological interpretability of our findings, we restricted our SMR analysis exclusively to cis-pQTLs. Following established protocols, cis-pQTLs were defined as significant SNPs (*p* < 5 × 10^−8^) located within a ±1 Mb window of the transcription start site (TSS). In the standard SMR test, the single most significantly associated *cis*-pQTL was utilized as the primary genetic instrument for each protein. Trans-pQTLs were intentionally excluded to avoid confounding pleiotropic effects. The Heterogeneity in Dependent Instruments (HEIDI) test was applied to distinguish true causality from linkage LD; associations with *P*_SMR_ < 0.05 and *P*_HEIDI_ > 0.05 were considered indicative of causality [26,27]. Subsequently, Bayesian colocalization was performed using the coloc R package (v4.6.3) to estimate the probability that GWAS and pQTL signals share a single causal variant, prioritizing targets with a posterior probability for Hypothesis 4 (*PP4*) > 0.5 [28,29]. To systematically prioritize candidates, we established an explicit three-tier classification: Tier 1 (High Confidence) required passing SMR (*p* < 0.05), HEIDI (*p* ≥ 0.05), and strong colocalization (*PP4* > 0.5); Tier 2 (Moderate Confidence) passed SMR but failed either HEIDI (suggesting potential horizontal pleiotropy) or strict colocalization; and Tier 3 (Low Confidence) passed PWAS but failed multiple causal/pleiotropy checks. To rule out reverse causality, we also performed a Reverse MR analysis using 32 independent genome-wide significant SNPs associated with POAG as instrumental variables to assess their causal effects on candidate plasma protein levels.

### 4.4. Single-Cell Transcriptomic Analysis & Functional Assessment

Single-cell RNA sequencing (scRNA-seq) data for human and mouse ocular tissues were obtained from published atlases [30]. We utilized the Single Cell Portal’s interactive tools to generate Uniform Manifold Approximation and Projection (UMAP) and dot plots, quantifying the expression intensity and cellular percentage of candidate genes across defined cell types. Concurrently, Gene Ontology (GO) and KEGG pathway enrichment analyses were executed via the clusterProfiler package (version 4.14.6) utilizing the org.Hs.eg.db annotation database (version 3.20.0) [31], and Protein–Protein Interaction (PPI) networks were constructed using the STRING (confidence score > 0.4) [32]. Target druggability was assessed using DGIdb 4.020 and DrugBank v5.1.13 [33].

## 5. Conclusions

This integrative multi-omics study unveils a landscape of prioritized candidate proteins for POAG, highlighting the convergence of ER stress (SEL1L) and endothelial regulation (TFPI) pathways in the pathogenesis of ocular hypertension. By resolving these signals to single-cell resolution within the outflow tract, we provide concrete mechanistic hypotheses and designate SEL1L and TFPI as priority targets for the development of next-generation glaucoma therapeutics.

## Figures and Tables

**Table 1:** Summary Results From PWAS, Colocalization, and SMR for 11 PWAS-Identified Proteins.

Protein	Protein Full Name	PWAS			Conditional		Colocalization	SMR			Category
		PWAS.Z	PWAS.P	PWAS.P.BH	JOINT.Z	JOINT.P	PP4 > 0.5	Beta_SMR	P_SMR	P_HEIDI_SMR	
RABEPK	Rab9 effector protein with kelch motifs	4.7434	2.10 × 10^−6^	0.0035133	4.7	2.10 × 10^−6^	YES	0.410725	8.91 × 10^−5^	3.18E-03	tier2
ADAMTS13	A disintegrin and metalloproteinase with thrombospondin motifs 13	4.493	7.02 × 10^−6^	0.00587223	4.5	7.00 × 10^−6^	NO	0.215376	8.15 × 10^−6^	4.24E-04	tier3
SEL1L	Protein sel-1 homolog 1	−4.2488	2.15 × 10^−5^	0.009828875	−4.2	2.10 × 10^−5^	YES	−0.182418	4.53 × 10^−5^	5.76 × 10^−1^	tier1
TFPI	Tissue factor pathway inhibitor	−4.229	2.35 × 10^−5^	0.009828875	−4.2	2.30 × 10^−5^	YES	−0.186363	2.57 × 10^−5^	1.74 × 10^−1^	tier1
TIMP3	Metalloproteinase inhibitor 3	−4.143	3.43 × 10^−5^	0.01147678	−4.1	3.40 × 10^−5^	NO	−0.101985	3.52 × 10^−5^	4.28 × 10^−3^	tier3
NFU1	NFU1 iron-sulfur cluster scaffold	3.9548	7.66 × 10^−5^	0.021358633	4	7.70 × 10^−5^	NO	0.676579	1.94 × 10^−4^	5.21 × 10^−2^	tier2
ITGA11	Integrin alpha-11	−3.8313	0.000127	0.030353	−3.8	0.00013	NO	−0.126206	6.75 × 10^−2^	3.45 × 10^−1^	tier3
SLC9A3R2	Na(+)/H(+) exchange regulatory cofactor NHE-RF2	−3.7282	0.000193	0.039966111	−3.7	0.00019	NO	−0.395208	1.47 × 10^−3^	1.92 × 10^−1^	tier2
SERPINF1	Pigment epithelium-derived factor	−3.701	0.000215	0.039966111	−3.7	0.00021	NO	−0.196203	2.32 × 10^−4^	1.36 × 10^−1^	tier2
MEGF10	Multiple epidermal growth factor-like domain protein 10	3.649	0.000263	0.042737545	3.6	0.00026	NO	0.132509	3.09 × 10^−4^	3.86 × 10^−4^	tier3
TP73	Tumor protein p73	3.632	0.000281	0.042737545	3.6	0.00028	NO	-	-	-	tier3

PP4 > 0.5 means 2 signals were considered to have a strong support of colocalization. PWAS.Z: Z-score from discovery PWAS analysis; PWAS.P: *p*-value from discovery PWAS analysis; PWAS.P.BH: *p*-value adjusted from discovery PWAS analysis; JOINT.Z: Z-score from conditional analysis; JOINT.P: *p*-value from conditional analysis; Beta_SMR: Effect estimate from the top snp SMR analysis; P_SMR: *p*-value from the top snp SMR analysis; P_HEIDI_SMR: *p*-value from HEIDI test.

## Data Availability

Publicly available datasets were analyzed in this study. These data can be found here: The GWAS summary statistics for POAG from the FinnGen Consortium (https://www.finngen.fi/en, accessed on 19 May 2026); the pQTL data from the UK Biobank Pharma Proteomics Project (https://registry.opendata.aws/ukbppp/, accessed on 19 May 2026); and the single-cell RNA sequencing data of ocular tissues (project number: SCP1845) can be accessed via the Broad Institute Single Cell Portal (https://singlecell.broadinstitute.org/single_cell, accessed on 19 May 2026).

## Supplementary Materials

The following supporting information can be downloaded at: https://www.mdpi.com/article/10.3390/ijms27114684/s1.

## Abbreviations

The following abbreviations are used in this manuscript:

DGIdb	Drug Gene Interaction Database
ECM	Extracellular matrix
ER	Endoplasmic reticulum
GO	Gene Ontology
GWAS	Genome-Wide Association Studies
HEIDI	Heterogeneity in Dependent Instruments
IOP	Intraocular pressure
JCT	Juxtacanalicular tissue
KEGG	Kyoto Encyclopedia of Genes and Genomes
LD	Linkage disequilibrium
MR	Mendelian Randomization
POAG	Primary open-angle glaucoma
PPI	Protein–Protein Interaction
pQTL	Proteomic quantitative trait loci
PWAS	Proteome-Wide Association Study
scRNA-seq	Single-cell RNA sequencing
SNP	Single Nucleotide Polymorphism
TFPI	Tissue factor pathway inhibitor
TM	Trabecular meshwork
UKB-PPP	UK Biobank Pharma Proteomics Project
